# Binocular Diplopia: A Possible Adverse Effect of Fluoroquinolone Therapy

**DOI:** 10.1155/2020/8843182

**Published:** 2020-11-24

**Authors:** Mariama Touray, Victoria Ando, Erwin Samutelela, Jean-Philippe Zuber

**Affiliations:** ^1^Etablissements Hospitaliers du Nord Vaudois, Internal Medicine, Saint-Loup Hospital, Pompaples, Switzerland; ^2^Jules-Gonin Ophthalmic Hospital, Lausanne, Switzerland

## Abstract

The adverse effects of fluoroquinolones are yet to be fully elucidated. We present an interesting case of a 41-year-old male with binocular diplopia most likely induced by the use of a fluoroquinolone antibiotic.

## 1. Introduction

Fluoroquinolones are widely used antibiotics with proven safety and efficacy (1, 2). However, adverse effects unique to this class of antibiotic, particularly tendinopathies, have been described. The mechanism underlying this adverse effect is not yet clear. This report presents a case of diplopia likely secondary to fluoroquinolone use.

## 2. Case Presentation

**Figure 1:**
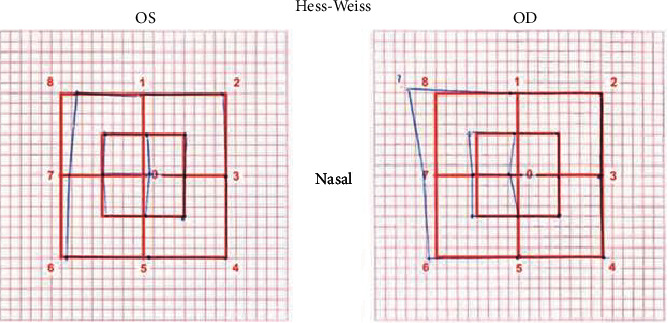
The Hess-Weiss test at presentation. Abbreviations: OS, oculus sinister, left eye; OD, oculus dexter, right eye.

A previously healthy 41-year-old male, with no known cardiovascular risk factors, attended the emergency department with painless horizontal binocular diplopia which had started 12 hours earlier. Three days prior to the onset of symptoms, the patient had been started on ciprofloxacin for a complicated urinary tract infection. The neuroophthalmologic examination was remarkable for the presence of horizontal binocular diplopia on leftward gaze. Visual acuity was 20/16 oculus uterque (OU) without correction. Intraocular tension was 12 mmHg OU. Slit lamp examination and fundoscopy were within normal limits OU. On the Hess-Weiss testing, we identified diminished function of the left *lateral rectus muscle* and an associated increased compensatory function of the right *medial rectus muscle*, confirming a paresis of the left *lateral rectus muscle* (Figure 1). The rest of the physical examination was unremarkable.

**Figure 2:**
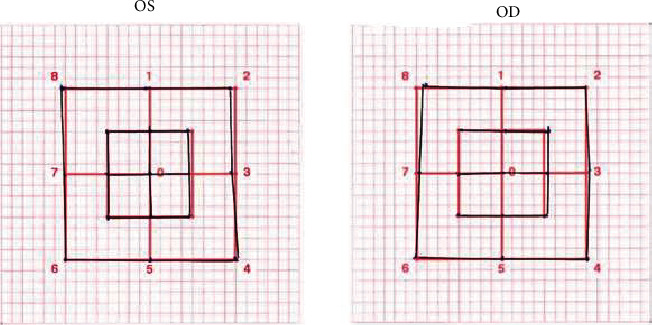
The Hess-Weiss test after ciprofloxacin cessation. Abbreviations: OS, oculus sinister, left eye; OD, oculus dexter, right eye.

Blood tests revealed a slightly increased CRP without leukocytosis. TSH was normal. Cerebral imaging (MRI and CT-angiography) and orbital MRI results were within normal limits. Analysis of cerebrospinal fluid was normal. Serology tests (HIV, syphilis, Lyme disease, and TBE) were negative. Taken together, we concluded on a probable fluoroquinolone-induced unilateral tendinopathy of the left abducens muscle. Ciprofloxacin was stopped and replaced by coamoxicillin. The patient reported progressive regression of diplopia after withdrawal of ciprofloxacin. At a follow-up consultation one week after ciprofloxacin withdrawal, the patient's symptoms had fully resolved. Three months later, the Hess-Weiss testing showed a complete recovery (Figure 2).

## 3. Discussion

Possible tendinitis of extraocular muscles due to fluoroquinolones has already been described in one 2009 database study (3). The study collected data on 171 patients who were enrolled in a national registry for pharmacological surveillance due to suspected fluoroquinolone-induced diplopia (3). Adverse events were noted at a median time of 9.6 days from the beginning of antibiotics, although time to onset ranged from 1 day to 5 months (3). Mean age at presentation was 51 years (3). Concomitant tendinopathy was found in 10% of patients (3).

Some risk factors are known to increase the occurrence of quinolone-induced tendinopathy, such as older age, chronic renal failure, concomitant steroid treatment, and systemic disease (4, 5). In the 2009 database study, a minority (29%) were 60 years or older, and 2% were on systemic steroids (3).

To our knowledge, only one other case report on this subject has been described in France (6) with a very similar clinical presentation to ours. The patient, a 58-year-old male with no known cardiovascular risk factors, had binocular diplopia due to paresis of the left abducens muscle arising during treatment with ofloxacin (6). The patient's diplopia reoccurred with the reintroduction of ofloxacin for a later infection, underscoring the correlation between diplopia and quinolone use (6).

Here, we present the case of a 41-year-old healthy male who presented with binocular diplopia 72 hours after introduction of fluoroquinolone therapy. Symptoms regressed rapidly after withdrawal of the culprit antibiotic, with complete remission noted at a follow-up consultation one week later.

## 4. Conclusion

The present case study demonstrates a correlation between the onset of diplopia after the initiation of fluoroquinolone therapy, and regression of the symptom upon withdrawal of this agent. However, fluoroquinolone-induced diplopia remains a diagnosis of exclusion. We encourage clinicians to consider discontinuing use of this antibiotic class when facing this possible diagnosis. The mechanism underlying the development of tendinopathies secondary to fluoroquinolone use is yet to be elucidated.

## Abbreviations

OU: Oculus uterque, both eyes
OS: Oculus sinister, left eye
OD: Oculus dexter, right eye
